# When the Electromyography (EMG) and the Patient Disagree: Unraveling a Motor Neuron Disease Mimic With Small-Fiber Dysfunction

**DOI:** 10.7759/cureus.115700

**Published:** 2026-09-03

**Authors:** Niyas Khalid Ottu Para

**Affiliations:** 1 Internal Medicine, Burjeel Hospital, Abu Dhabi, ARE

**Keywords:** amyotrophic lateral sclerosis, electromyography, fasciculations, motor neuron disease mimic, peripheral nerve hyperexcitability, radiculopathy, small-fiber dysfunction, sudomotor dysfunction

## Abstract

Amyotrophic lateral sclerosis (ALS) is a progressive neurodegenerative disorder characterized by upper and lower motor neuron dysfunction. Electromyography (EMG) is central to its evaluation, but neurogenic abnormalities and fasciculations are not specific to ALS and must be interpreted within the clinical phenotype and disease trajectory. This study reports a 52-year-old man with an approximately eight-year history of intermittent muscle twitching, bilateral upper-limb tremor, muscular tightness and warmth, and asymmetric cutaneous hypersensitivity. An initial electrodiagnostic evaluation raised concern for diffuse motor neuron disease, despite preserved strength, normal gait, absence of muscle wasting, and lack of clear upper motor neuron signs. Extensive evaluation for inflammatory, autoimmune, metabolic, toxic, infectious, paraneoplastic, neuromuscular junction, and peripheral nerve hyperexcitability disorders was unrevealing. Serum creatine kinase was mildly and variably elevated, while aldolase and a comprehensive myositis panel were normal. Brain magnetic resonance imaging (MRI) demonstrated no supportive features of ALS. Sudomotor testing showed reduced electrochemical skin conductance (ESC) in the hands, supporting small-fiber/autonomic dysfunction in the context of prominent sensory symptoms. Repeat EMG, performed after interval follow-up with broad muscle sampling, demonstrated focal neurogenic abnormalities involving bilateral ulnar distributions and left L5-S1 radiculopathy but found no electrophysiological evidence of a neurodegenerative disorder. The patient was treated symptomatically with gabapentin and vitamin E and reported approximately 80% improvement after six weeks. This case highlights the importance of clinicophysiological correlation, disease trajectory, sensory phenomenology, and repeat electrophysiology when an initial EMG suggests motor neuron disease but the longitudinal clinical picture is discordant. It also illustrates how small-fiber dysfunction and focal neurogenic abnormalities may coexist and mimic a progressive motor neuron syndrome.

## Introduction

Amyotrophic lateral sclerosis (ALS) is a progressive neurodegenerative disorder characterized by degeneration of upper and lower motor neurons, ultimately resulting in progressive weakness, muscle wasting, bulbar dysfunction, respiratory compromise, and death. Despite advances in neuroimaging, electrophysiology, biomarkers, and genetics, ALS remains fundamentally a clinical diagnosis supported by neurophysiological findings and the exclusion of alternative disease processes. Contemporary diagnostic frameworks, including the Gold Coast criteria, emphasize the presence of progressive motor impairment, documented by history or repeated clinical assessment, together with evidence of upper and/or lower motor neuron dysfunction and appropriate exclusion of alternative diagnoses [1]. Electromyography (EMG) plays a central supportive role by identifying active denervation and chronic neurogenic change, particularly when lower motor neuron involvement is not yet clinically apparent; however, electrophysiological abnormalities cannot be interpreted independently of the clinical phenotype and longitudinal disease course [1].

This distinction becomes particularly important in patients presenting with fasciculations. Although fasciculations are strongly associated with motor neuron disease in clinical practice, they are not disease-specific and occur in a broad spectrum of conditions including benign fasciculation syndrome (BFS), cramp-fasciculation syndrome (CFS), peripheral nerve hyperexcitability, radiculopathies, focal neuropathies, metabolic disturbances, and other peripheral nerve disorders. Importantly, minor chronic neurogenic abnormalities may coexist with benign fasciculations without indicating evolving ALS. In a longitudinal study of patients evaluated because of concern for ALS, Montalvo et al. demonstrated that some patients with BFS had chronic neurogenic potentials in addition to fasciculations on EMG; these electrophysiological abnormalities remained stable during prolonged follow-up, and the majority of patients experienced symptomatic improvement rather than progression to motor neuron disease [2].

Discordance between electrophysiological findings and the clinical phenotype should prompt diagnostic reassessment rather than automatic escalation toward ALS. Because progressive motor impairment is intrinsic to contemporary ALS diagnostic frameworks, a prolonged history of involuntary muscle phenomena without objective weakness, wasting, bulbar dysfunction, gait deterioration, or emerging upper motor neuron signs should prompt careful consideration of alternative diagnoses [1].

Classical ALS remains predominantly a motor syndrome, whereas symptoms such as burning, abnormal warmth, hyperesthesia, allodynia, and dysesthesia raise the possibility of peripheral sensory involvement. Small-fiber dysfunction is particularly relevant because thinly myelinated Aδ fibers and unmyelinated C fibers mediate nociceptive and thermal sensation as well as several autonomic functions. Conventional nerve conduction studies predominantly interrogate large myelinated fibers and may therefore remain normal despite clinically significant small-fiber pathology. Current diagnostic approaches to small-fiber neuropathy (SFN) consequently emphasize integration of the clinical phenotype with dedicated functional or structural assessments such as quantitative sensory testing (QST) and intraepidermal nerve-fiber density (IENFD) on skin biopsy [3]. A recent 2026 evidence-based review from the American Association of Neuromuscular and Electrodiagnostic Medicine (AANEM) similarly emphasized structured evaluation for potentially identifiable metabolic and immune etiologies in patients with suspected SFN [4].

Sudomotor assessment may provide additional supportive evidence of small-fiber/autonomic dysfunction. Electrochemical skin conductance (ESC), measured using Sudoscan (SUDOSCAN 2; Impeto Medical, Paris, France), has been investigated as a rapid non-invasive method of evaluating sweat gland-related function and has been studied across several peripheral neuropathic disorders [5,6]. However, its diagnostic role remains debated, and ESC abnormalities should not be considered equivalent to histological confirmation of SFN. Systematic reviews have highlighted heterogeneity in diagnostic performance and limitations in establishing ESC as a standalone measure of sensory or autonomic small-fiber integrity [6,7]. Thus, an abnormal Sudoscan result is most appropriately interpreted in conjunction with compatible sensory symptoms and other clinical or neurophysiological findings.

Against this background, the present study reports a diagnostically challenging case in which electrophysiological findings initially raised concern for motor neuron disease but were discordant with the clinical phenotype and an unusually prolonged course without objective motor progression. This case underscores the importance of integrating electrophysiological findings with disease trajectory and clinical phenotype, particularly when an abnormal EMG appears inconsistent with the expected evolution of a progressive neurodegenerative disorder.

## Case presentation

A 52-year-old man with a background of hyperlipidemia and cervical degenerative disc disease was evaluated for persistent involuntary muscle movements involving the upper and lower extremities. His initial presentation had raised considerable concern for motor neuron disease because of widespread muscle twitching and an abnormal electrophysiological study. However, detailed reassessment of his clinical history subsequently revealed a considerably longer and more complex symptom trajectory than was apparent from the initial neurological evaluation.

The patient recalled that his symptoms had first begun approximately eight years earlier, during a period of profound emotional stress after his daughter was involved in a serious accident. Over subsequent years, he experienced intermittent abnormal muscular sensations and involuntary movements without progressive loss of physical function. More recently, the symptoms had become sufficiently prominent to prompt neurological investigation. He described recurrent twitching involving different muscle groups, together with a persistent subjective sensation that his muscles were unusually tense and warm. He also reported bilateral upper-limb tremor, more prominent on the left, which he confirmed had been present for several years. In addition to the motor phenomena, he described distinctly sensory symptoms, particularly asymmetric hypersensitivity of the skin. Importantly, he repeatedly denied true muscular weakness. He remained independently ambulant and physically active, reported no deterioration in gait or functional capacity, and specifically noted that exercise tended to relieve rather than exacerbate the sensation of muscular tension. He had no other chronic medical conditions, was a former smoker, and was not an alcoholic.

There was also a remote neurological history. Approximately 22 years earlier, he had experienced lower back pain radiating into the left lower extremity while playing basketball, associated with transient weakness and subsequent atrophy of the left calf. The weakness had subsequently improved. This history later became relevant when chronic lumbosacral neurogenic abnormalities were identified electrophysiologically.

During the neurological assessment preceding his referral, fasciculations had been observed in the left triceps and intrinsic hand muscles, more prominently on the left, occasionally producing visible movement of the thumb. Motor power was documented as normal overall, although mild weakness of the left ankle flexors was noted during calf-raise testing. Reflexes were generally preserved, with the exception of an absent left ankle jerk. No convincing upper motor neuron syndrome was documented. In particular, there was no reported spasticity, pathological hyperreflexia, or other clear pyramidal manifestation. During subsequent clinical assessment, the involuntary upper-limb movements appeared predominantly tremulous rather than representing continuous widespread fasciculations. The tremor remained bilateral and asymmetric, with greater involvement of the left upper extremity. Muscle power and gait were preserved, and there was no clinically apparent progressive muscle wasting or functional motor deficit.

Initial nerve conduction studies demonstrated preserved bilateral median and ulnar sensory responses, normal bilateral sural sensory responses, and preserved motor conduction in the median, ulnar, peroneal, and tibial nerves. No conduction block was demonstrated. Needle EMG examination, however, identified active and chronic neurogenic abnormalities in selected muscles. The left gastrocnemius demonstrated fibrillation potentials, positive sharp waves, and complex repetitive discharges (CRDs), while the left triceps and bilateral first dorsal interosseous muscles showed fasciculation potentials and large, unstable motor-unit potentials with reduced neurogenic recruitment. On the basis of these findings, the study was interpreted as being consistent with diffuse motor neuron disease, with repeat electrophysiological assessment after three to six months recommended for diagnostic confirmation.

The electrophysiological interpretation generated concern for ALS. However, several elements of the clinical phenotype appeared discordant with this diagnosis. Most notably, the patient described an approximately eight-year symptom history without progressive objective weakness, gait deterioration, bulbar dysfunction, or substantial loss of motor function. Sensory hypersensitivity was prominent, and muscular tightness improved with exercise. These discrepancies prompted a systematic search for alternative neuromuscular, inflammatory, autoimmune, metabolic, toxic, infectious, and paraneoplastic explanations rather than accepting the initial electrophysiological impression as definitive.

MRI of the cervical spine demonstrated multilevel degenerative spondylotic disease with foraminal narrowing and nerve-root involvement at several levels but no significant spinal cord compression or myelopathic signal abnormality. Lumbar MRI demonstrated multilevel degenerative disc disease, most pronounced at L4-L5 and L5-S1, including disc desiccation and loss of disc height at L5-S1, without significant central canal stenosis or definite nerve-root impingement (Figure 1). These findings provided anatomical context for the chronic left L5-S1 abnormalities and were subsequently demonstrated electrophysiologically; however, these were insufficient to explain the patient's generalized symptom complex.

**Figure 1:**
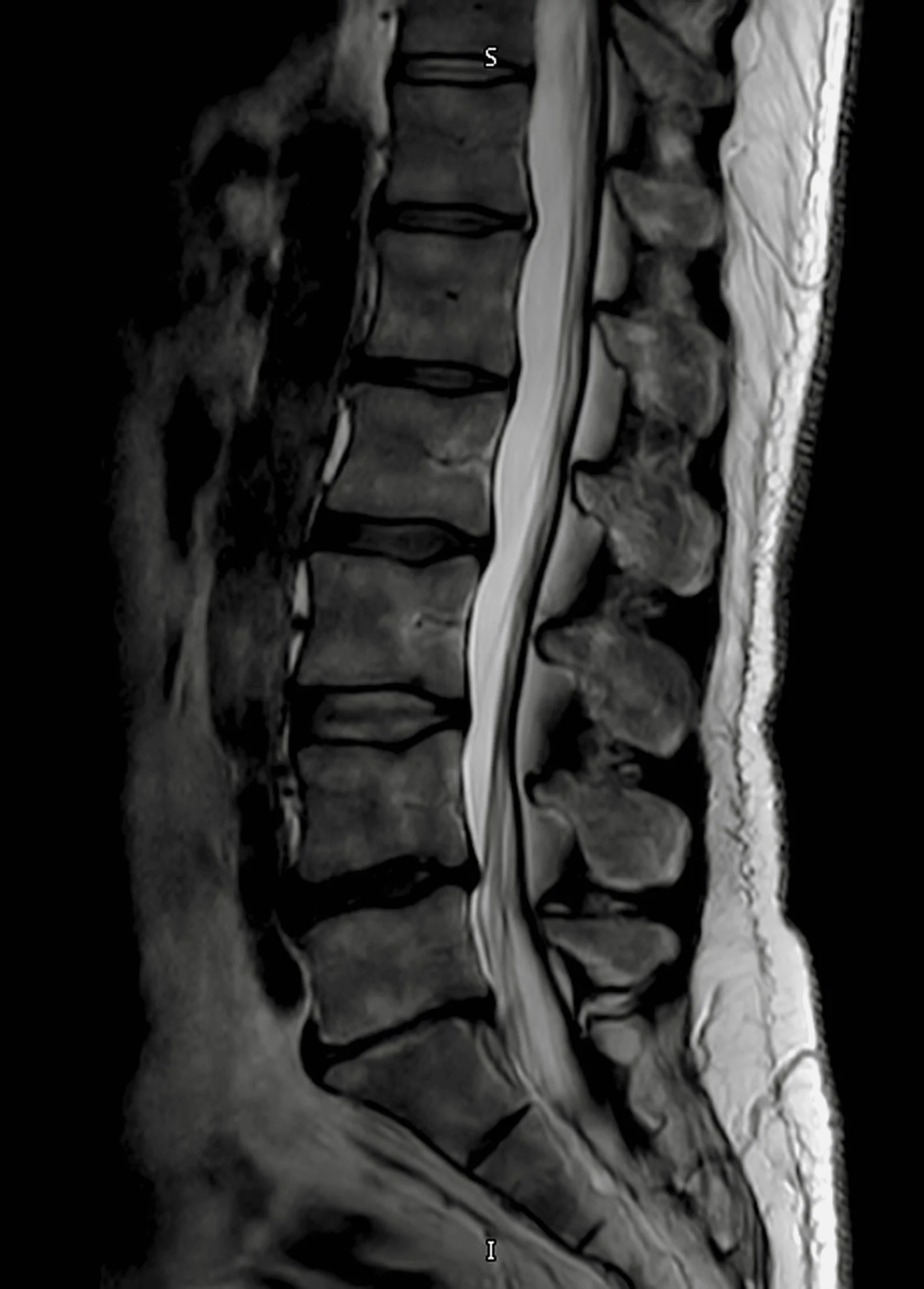
Magnetic resonance imaging of the lumbar spine Sagittal T2-weighted magnetic resonance image demonstrating multilevel degenerative disc disease, most pronounced at L4-L5 and L5-S1, with disc desiccation and loss of disc height at L5-S1. No significant central canal stenosis was identified.

Given the concern for a central motor disorder, MRI of the brain with contrast was subsequently obtained. There was no abnormal signal along the corticospinal tracts, no convincing motor-band sign involving the precentral gyri, no brainstem or cerebellar lesion, and no pathological intracranial enhancement. Minimal punctate susceptibility foci were described in the bilateral precentral gyri, possibly reflecting mild ferritin deposition, but were considered of uncertain clinical significance. The radiological conclusion specifically noted the absence of definitive MRI features suggestive of ALS (Figure 2).

**Figure 2:**
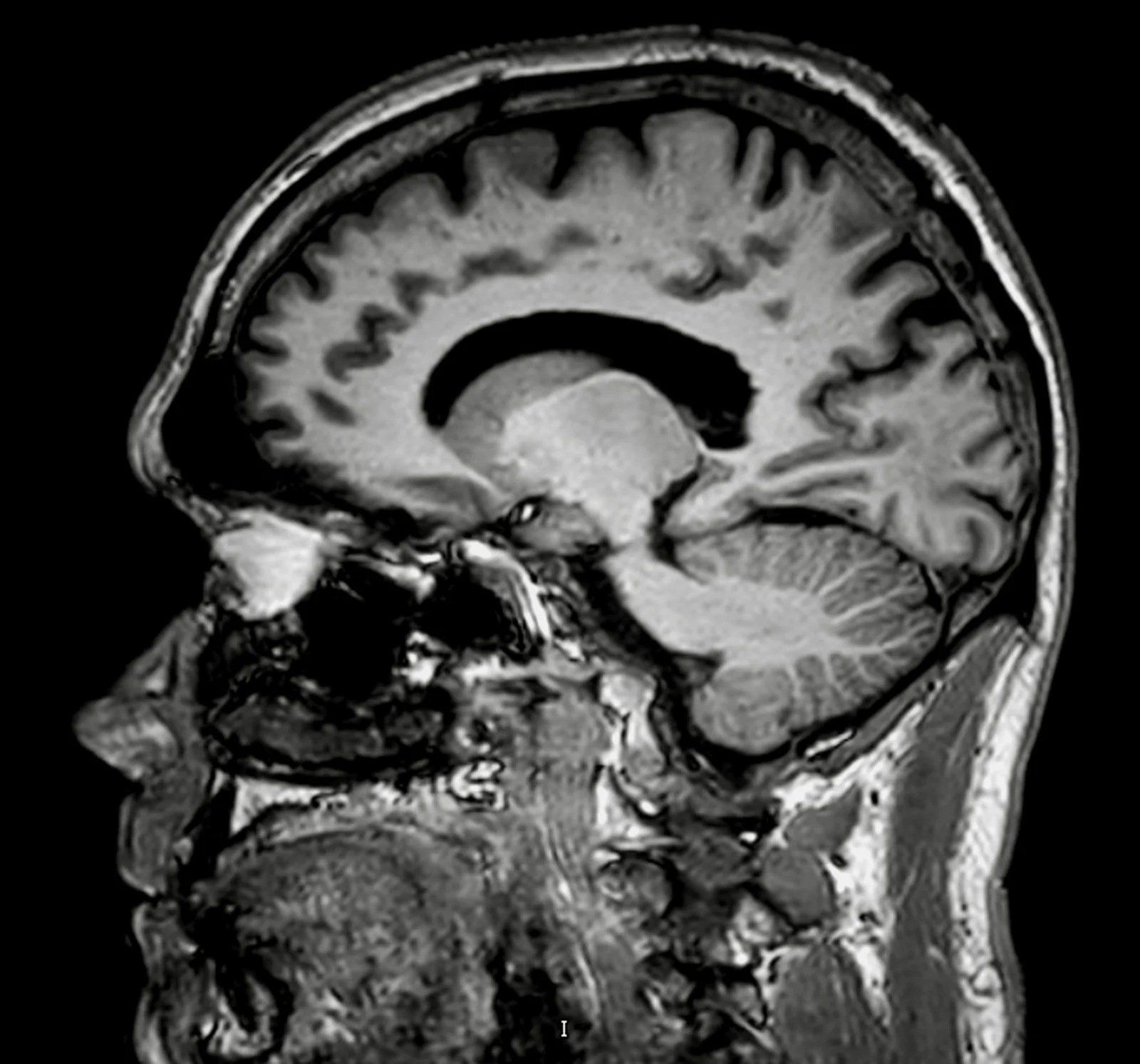
Magnetic resonance imaging of the brain Sagittal T1-weighted magnetic resonance image demonstrating preserved cerebral and brainstem morphology without a structural lesion to account for the patient's neurological presentation. The complete MRI examination demonstrated no definitive imaging features supportive of amyotrophic lateral sclerosis (ALS).

Serum creatine kinase was mildly elevated at 659 U/L during the initial investigation. Repeat measurements demonstrated fluctuation rather than a progressive rise, decreasing to 382 U/L and subsequently measuring 549 U/L. Aldolase was normal. In the absence of objective proximal weakness or a myopathic electrophysiological pattern, the modest hyperCKemia was not considered sufficient to establish a primary muscle disorder. A comprehensive myositis antibody panel was negative, including antibodies directed against Jo-1, PL-7, PL-12, EJ, OJ, Mi-2α, Mi-2β, MDA5, NXP2, TIF1-γ, SAE1, SRP, PM-Scl75, PM-Scl100, Ku, Ro52, cN-1A, Ha, and Zo.

A broad autoimmune evaluation was undertaken because of the sensory manifestations and associated cutaneous changes. Antinuclear antibody testing was borderline positive at approximately 1:100; however, anti-double-stranded DNA antibodies and the extractable nuclear antigen panel were negative, complement C3 and C4 concentrations were normal, and anti-cyclic citrullinated peptide antibodies were negative. Both p-antineutrophil cytoplasmic antibody (ANCA) and c-ANCA were negative. Lupus anticoagulant and anticardiolipin antibodies were negative. An isolated elevation of β2-glycoprotein I IgM was identified; however, repeat testing was negative, and there were insufficient clinical or laboratory criteria to establish antiphospholipid syndrome.

Investigation for disorders of neuromuscular transmission and immune-mediated peripheral nerve hyperexcitability was similarly unrevealing. Acetylcholine receptor antibodies were negative, as were GAD65 antibodies and voltage-gated potassium channel antibodies. A broad paraneoplastic neuronal antibody panel was negative. Testing for additional metabolic, toxic, and infectious mimics included normal lactate and pyruvate, normal lactate dehydrogenase, normal cortisol, and a negative heavy-metal screen. Serological testing for human T-cell lymphotropic virus (HTLV) and hepatitis C was negative. Collectively, these investigations provided no convincing evidence of an inflammatory myopathy, systemic connective-tissue disease, recognized antibody-mediated peripheral nerve hyperexcitability syndrome, neuromuscular junction disorder, toxic neuropathy, metabolic myopathy, or paraneoplastic neurological syndrome. The major investigations are summarized in Table 1.

**Table 1:** Pertinent laboratory investigations during diagnostic evaluation ANA: antinuclear antibody; ANCA: antineutrophil cytoplasmic antibody; ESR: erythrocyte sedimentation rate; GAD: glutamic acid decarboxylase; HTLV: human T-cell lymphotropic virus; LDH: lactate dehydrogenase; TSH: thyroid-stimulating hormone; PCNA: proliferating cell nuclear antigen; nRNP: nuclear ribonucleoprotein; Mi-2: Mi-2 antibody; Ku: Ku antibody; PM-Scl: polymyositis-scleroderma; Ro52: Ro52 antibody; CCP: cyclic citrullinated peptide; IFA: indirect immunofluorescence assay; ANA: antinuclear antibody; eGFR: estimated glomerular filtration rate; WBC: white blood cell; HbA1c: glycated hemoglobin A1c; AST: aspartate aminotransferase; ALT: alanine aminotransferase

Parameter	Patient value	Reference range/interpretation
Hematologic and inflammatory evaluation		
Hemoglobin	14.3 g/dL	13-17 g/dL
WBC count	6.02 × 10⁹/L	4-10 × 10⁹/L
Platelet count	271 × 10⁹/L	150-410 × 10⁹/L
ESR	6 mm/h	0-15 mm/h
Muscle and metabolic evaluation		
Creatine kinase (CK), initial	659 U/L	0-190 U/L
CK, repeat	382 U/L	0-190 U/L
CK, subsequent	549 U/L	0-190 U/L
Lactate	0.9-1.0 mmol/L	0.5-2.2 mmol/L
LDH	185 U/L	135-225 U/L
AST	35 U/L	0-40 U/L
ALT	42 U/L	0-41 U/L
Creatinine	100 µmol/L	62-106 µmol/L
eGFR	79 mL/min/1.73 m²	>90 mL/min/1.73 m²
Sodium	136 mmol/L	136-145 mmol/L
Potassium	4.3 mmol/L	3.7-5.5 mmol/L
Magnesium	0.854 mmol/L	0.81-1.45 mmol/L
Phosphorus	1.30 mmol/L	0.660-1.070 mmol/L
Total calcium	2.38 mmol/L	2.15-2.50 mmol/L
Ionized calcium	1.00 mmol/L	1.17-1.29 mmol/L
Vitamin B12	487 pg/mL	211-946 pg/mL
25-hydroxyvitamin D	39 ng/mL	30-80 ng/mL
TSH	1.54 mIU/L	0.270-4.200 mIU/L
HbA1c	5.70%	4.0-5.7%
Autoimmune and inflammatory evaluation		
ANA by IFA	Borderline, minimal fluorescence of low significance	1:100
Anti-dsDNA antibody	10 IU/mL	0-100 IU/mL
Anti-CCP antibody	8 U/mL	≤17 U/mL
C3 complement	1.5 g/L	0.9-1.8 g/L
C4 complement	0.3 g/L	0.1-0.4 g/L
p-ANCA	Negative	Negative
c-ANCA	Negative	Negative
Anti-Ro52	Negative	Negative
Anti-PM-Scl	Negative	Negative
Anti-Ku	Negative	Negative
Anti-Mi-2	Negative	Negative
Anti-nRNP	Negative	Negative
Anti-PCNA	Negative	Negative
Anti-histone antibody	Negative	Negative
Comprehensive myositis antibody panel	Negative	Negative
Antiphospholipid evaluation		
Lupus anticoagulant	Negative on both assessments	Negative
Anticardiolipin IgG	Negative	<1.6
Anticardiolipin IgM	Negative	<12.5
β2-glycoprotein I IgG/IgM	β2-glycoprotein I IgM Initially elevated; repeat negative (transient positivity)	APS criteria not fulfilled
Neuromuscular/neurologic evaluation		
Acetylcholine receptor antibodies	<0.07 nmol/L	0.00-0.24 nmol/L
GAD autoantibody	1.7 IU/mL	<10 IU/mL
Paraneoplastic autoantibody panel	Negative	Negative
Infectious evaluation		
Anti-hepatitis C antibody	Non-reactive	Non-reactive
Anti-HTLV antibody	Non-reactive	Non-reactive

The patient's persistent sensory complaints prompted assessment of small-fiber and autonomic function. ESC was assessed using Sudoscan. Hand conductance measured 57 μS on the left and 61 μS on the right, falling within the device-reported range for moderately reduced sudomotor function, whereas foot conductance was relatively preserved at 76 μS on the left and 77 μS on the right, respectively. In the context of asymmetric cutaneous hypersensitivity, abnormal sensations of warmth, preserved large-fiber sensory nerve conduction, and the absence of objective motor deterioration, the finding was considered supportive of a small-fiber/sudomotor component to his symptom complex. Because confirmatory IENFD assessment was not performed, the finding was interpreted as small-fiber dysfunction rather than biopsy-confirmed SFN.

The discrepancy between the original EMG interpretation and the patient's clinical course remained the principal diagnostic concern. Rather than establishing a diagnosis of ALS on the basis of the initial study, repeat electrophysiological assessment was therefore arranged after an appropriate interval. The repeat examination was substantially broader, sampling 28 muscles across multiple anatomical distributions. Twenty-one of the muscles examined were electrophysiologically normal. Abnormal spontaneous or insertional activity was confined to the left L5 paraspinal muscles, while selected chronic neurogenic motor-unit abnormalities were predominantly localized to muscles within bilateral ulnar distributions. The findings were interpreted as neurogenic changes affecting muscles supplied by the bilateral ulnar nerves together with left L5-S1 radiculopathy. Crucially, the repeat electrophysiological report concluded that there was no electrophysiological evidence of a neurodegenerative disorder. A detailed comparison of the initial and repeat electrophysiological findings is presented in Table 2.

**Table 2:** Comparison of the initial and repeat electrophysiological studies Numerical reference ranges are reported only where provided in the original electrophysiological reports; findings otherwise designated as normal or abnormal reflect the interpreting neurophysiologist's assessment. CRD: complex repetitive discharge; EMG: electromyography; FDI: first dorsal interosseous; Fib: fibrillation potentials; MUAP: motor unit action potential; NCS: nerve conduction study; PPP: polyphasic potentials; PSW: positive sharp waves

Parameter	Initial electrophysiological study	Repeat needle EMG
Study scope	Initial NCS/needle EMG, abnormalities reported in selected upper- and lower-limb muscles.	Needle EMG of 28 muscles, 21 muscles normal.
Sensory NCS	Median velocities 68/67 m/s, ulnar 66/64 m/s, sural 57/55 m/s, interpreted as normal.	Not the central abnormality in the repeat report provided.
Motor NCS	Bilateral median, ulnar, peroneal, and tibial motor studies interpreted as normal; no conduction block reported.	Repeat report centered on needle EMG findings.
Left gastrocnemius	Fib 2+, PSW 2+, CRD present; MUAP/recruitment otherwise normal.	No active denervation/fasciculations, reduced recruitment.
Left triceps	Fasciculations 1+; large/unstable MUAP up to ~4 mV.	Normal.
Left FDI	Fasciculations 1+; large/unstable MUAP up to ~12 mV; reduced recruitment.	Giant MUAP and reduced recruitment, no active denervation/fasciculations.
Right FDI	Fib 1+, PSW 1+, fasciculations 1+; large/unstable MUAP up to ~12 mV; reduced recruitment.	Giant MUAP, no active denervation/fasciculations.
Paraspinal sampling	Not reported in available initial study.	Left L5 paraspinal Fib 2+, right L5 and right T9 paraspinals without active spontaneous activity.
Distribution	Multifocal selected abnormalities interpreted as concerning for diffuse motor neuron disease.	Focal bilateral ulnar-distribution neurogenic changes plus left L5-S1 root-distribution abnormalities.
Formal conclusion	Diffuse motor neuron disease suspected/considered; repeat study in three to six months recommended for confirmation.	No electrophysiological evidence for neurodegenerative disorders noted.
Longitudinal significance	Required interval confirmation.	Extensive repeat study did not confirm a diffuse progressive motor-neuron pattern.

With the combination of prolonged clinical stability, preserved power and gait, absence of convincing upper motor neuron manifestations, prominent sensory hypersensitivity, abnormal sudomotor testing, extensive negative investigation for secondary neuromuscular disorders, and repeat electrophysiology failing to confirm a generalized motor neuron process, the working diagnosis was revised toward a small-fiber-predominant neuropathic syndrome with coexisting focal neurogenic abnormalities and chronic radiculopathy rather than ALS. The chronological summary of the clinical course is summarized in Table 3.

**Table 3:** Chronological summary of the clinical course and diagnostic reassessment NCS: nerve conduction study; EMG: electromyography; ESC: electrochemical skin conductance; ALS: amyotrophic lateral sclerosis

Time	Clinical event	Key findings
Eight years before current evaluation	Symptom onset	Intermittent twitching, abnormal muscular sensations, tremor, and sensory hypersensitivity without progressive weakness
Subsequent years	Persistent intermittent symptoms	Preserved gait, strength, independence, and functional capacity
Initial neurological evaluation	NCS/EMG performed	Selected neurogenic abnormalities raised concern for diffuse motor neuron disease/ALS
Subsequent evaluation	Imaging and extensive laboratory assessment	No convincing inflammatory, autoimmune, toxic, infectious, metabolic, paraneoplastic, or neuromuscular-junction explanation
Sudomotor assessment	Electrochemical skin conductance testing	Reduced hand ESC with relatively preserved foot values, supportive of small-fiber/autonomic dysfunction
Repeat EMG	28 muscles sampled	21 normal, localized bilateral ulnar and left L5-S1 abnormalities, no electrophysiological evidence of a neurodegenerative disorder
Follow-up after treatment	Clinical reassessment	Patient-reported ~80% symptomatic improvement without new weakness or functional deterioration

The patient was commenced on gabapentin 300 mg daily for symptomatic treatment of the neuropathic and hyperexcitability manifestations, together with vitamin E 400 mg twice daily. Approximately six weeks after treatment initiation, he subjectively estimated an overall improvement of approximately 80%, describing substantial relief of the previously persistent abnormal muscular and sensory sensations and marked improvement in quality of life. This improvement represented the patient's overall subjective assessment rather than a validated symptom score. No treatment-related adverse effects were reported. At approximately two months of follow-up, the improvement remained sustained, with no new weakness, gait impairment, or functional neurological deterioration. Continuation of gabapentin and vitamin E with longitudinal clinical follow-up was therefore advised.

The therapeutic response was considered supportive of a clinically relevant neuropathic or neuronal-hyperexcitability component, although it was not regarded as independently diagnostic of SFN. More importantly, the combination of the patient's prolonged nonprogressive clinical course and the repeat electrophysiological findings ultimately resolved the principal diagnostic concern raised by the initial EMG and provided objective evidence against an evolving generalized motor neuron disorder.

## Discussion

The present case illustrates a diagnostically challenging scenario in which electrophysiological findings initially raised concern for diffuse motor neuron disease, while the patient's longitudinal clinical phenotype repeatedly argued against a progressive neurodegenerative motor disorder. The eventual resolution of this discordance required neither a single definitive biomarker nor an exotic diagnosis, but rather careful reconstruction of the disease timeline, recognition of sensory and autonomic features that were poorly explained by ALS, systematic exclusion of important mimics, and, critically, repeat electrophysiological assessment. The case therefore highlights a fundamental principle of neuromuscular medicine, that is, electrodiagnostic findings acquire diagnostic meaning only when interpreted within the clinical phenotype, anatomical distribution, and temporal evolution of disease.

ALS is fundamentally a progressive clinical syndrome. The Gold Coast criteria require progressive motor impairment documented by history or repeated clinical assessment, together with upper and/or lower motor neuron dysfunction and exclusion of alternative disease processes [1]. EMG is exceptionally valuable for demonstrating lower motor neuron involvement, particularly when denervation is not yet clinically obvious, but it remains an extension of the neurological examination rather than an independent diagnostic arbiter. Turner and the UK Motor Neuron Disease Clinical Studies Group specifically emphasized this relationship when discussing the Gold Coast criteria and the role of EMG in ALS diagnosis [1].

This distinction was particularly relevant in our patient because the natural history was strikingly discordant with ALS. Although his recent increase in muscle twitching had triggered neurological evaluation, detailed history subsequently established that involuntary muscular and sensory symptoms had been present intermittently for approximately eight years. During this prolonged interval, he had not developed progressive objective weakness, clinically meaningful muscle wasting, gait deterioration, bulbar dysfunction, respiratory impairment, or loss of functional independence. On repeated assessment, power and gait remained preserved, and there was no convincing upper motor neuron syndrome. The absence of spasticity alone would not exclude ALS, particularly an initially lower-motor-neuron-predominant phenotype; however, the combination of an eight-year history with preserved motor function fundamentally weakened the biological plausibility of an actively progressive ALS phenotype.

The distinction between fasciculation and progressive motor neuron degeneration is similarly important. Fasciculations are conspicuous and understandably alarming, but they are not specific to ALS [2]. They occur in BFS, CFS, radiculopathies, focal neuropathies, peripheral nerve hyperexcitability states, metabolic disturbances, and even otherwise healthy individuals. Particularly relevant to the present case is the longitudinal study by Montalvo et al., in which 37 patients with BFS were evaluated, many following referral because of suspected ALS. Seven demonstrated chronic neurogenic potentials in addition to fasciculation potentials on their initial EMG. Among patients undergoing longitudinal electrophysiological assessment, including those with baseline neurogenic abnormalities, these findings remained stable, and approximately two-thirds of the cohort reported symptomatic improvement. The authors concluded that minor EMG abnormalities in patients with benign fasciculations do not necessarily predict progression to ALS [2].

This observation provides an important conceptual parallel to our patient. His initial study was genuinely abnormal and should not retrospectively be dismissed as a technically meaningless result. Active and chronic neurogenic abnormalities were demonstrated in selected muscles, including the left gastrocnemius, left triceps, and bilateral first dorsal interosseous muscles. The crucial question, however, was whether these abnormalities represented a diffuse progressive anterior horn cell process or whether they could be explained by a combination of chronic radiculopathic, focal neurogenic, and hyperexcitability phenomena. The subsequent clinical course and repeat EMG strongly favored the latter interpretation.

The repeat electrophysiological examination was therefore the pivotal investigation in this case. Broad sampling of 28 muscles demonstrated that 21 were electrophysiologically normal. Abnormal spontaneous activity was localized rather than generalized, and the remaining neurogenic changes were predominantly distributed within bilateral ulnar territories and the left L5-S1 distribution. Most importantly, the interpreting neurophysiologist found no electrophysiological evidence of a neurodegenerative disorder. Rather than demonstrating progressive dissemination of active and chronic denervation into additional anatomical regions, the repeat study anatomically localized the abnormalities. This pattern was much more concordant with the patient's preserved motor function and known radiculopathic history than with progressive generalized motor neuron degeneration.

This case consequently demonstrates why repeat EMG can provide information that is qualitatively different from merely repeating an abnormal test. In a progressive motor neuron disorder, time is expected to reveal biological progression: objective weakness develops, denervation spreads, additional anatomical regions become involved, and the clinicophysiological pattern becomes increasingly coherent. Conversely, stability or localization of electrophysiological abnormalities, particularly when accompanied by preserved motor function, should prompt reconsideration of the original diagnostic framework [1,2]. The diagnostic value of the second EMG in our patient therefore lay not simply in producing a different conclusion but in testing the hypothesis of progression.

Sensory phenotype and the small-fiber hypothesis

The patient's sensory phenotype represented another major clue against a purely motor disorder. He consistently described asymmetric cutaneous hypersensitivity together with abnormal sensations of muscular warmth and tension. These symptoms were not accompanied by large-fiber sensory abnormalities on conventional nerve conduction studies. Rather than considering this discrepancy contradictory, it raised the possibility that the symptomatic fibers were not being adequately interrogated by routine NCS.

SFN affects thinly myelinated Aδ fibers and unmyelinated C fibers. These fibers mediate thermal sensation, nociception, and several autonomic functions. Their dysfunction can therefore produce positive sensory phenomena including burning, abnormal warmth, hyperalgesia, allodynia, and dysesthesia, as well as autonomic manifestations [3]. Importantly, conventional nerve conduction studies predominantly evaluate large myelinated fibers and may remain normal in isolated or predominantly small-fiber disease [3].

The diagnostic difficulty of SFN arises precisely from this clinicophysiological dissociation. Devigili et al. demonstrated that robust diagnosis requires integration of clinical signs with objective small-fiber measures, particularly QST and IENFD [3]. Skin biopsy with quantification of IENFD is an established objective technique for evaluating small-fiber pathology, with the European Federation of Neurological Societies/Peripheral Nerve Society guideline supporting distal-leg biopsy as a reliable diagnostic method [8].

Our patient did not undergo IENFD assessment, and this represents an important limitation. Accordingly, we deliberately describe his condition as small-fiber dysfunction rather than biopsy-confirmed SFN. Nevertheless, several features converged toward small-fiber involvement: prominent positive sensory symptoms, cutaneous hypersensitivity, abnormal perceptions of warmth, preserved conventional sensory nerve conduction, and abnormal sudomotor testing [3,9]. The recent 2026 AANEM Small Fiber Neuropathy Task Force evidence-based review further emphasizes the importance of structured investigation for potentially identifiable etiologies in patients with suspected SFN and reflects the increasing recognition of this heterogeneous group of disorders in contemporary neuromuscular practice [4].

Interpreting the Sudoscan finding

ESC testing provided an additional objective clue. The patient demonstrated reduced conductance in the hands with relatively preserved values in the feet. Sudoscan measures ESC related to sweat-gland function and has been proposed as a rapid, non-invasive method of assessing sudomotor dysfunction [5,6]. Because eccrine sweat glands receive sympathetic cholinergic innervation through unmyelinated fibers, abnormal sudomotor function can provide indirect evidence of small autonomic fiber dysfunction [10].

However, this finding requires appropriate restraint. ESC is not synonymous with IENFD measurement and does not independently establish SFN [6,7]. Novak's systematic review of 24 studies concluded that ESC expands the available methods for evaluating sudomotor dysfunction but also emphasized differences between ESC and established autonomic/small-fiber techniques and the need to interpret its diagnostic performance within those limitations [6].

Accordingly, the abnormal Sudoscan result in our patient should not be viewed as the single test that replaced an ALS diagnosis with SFN. Its importance lies instead in clinicophysiological concordance. The patient had prominent sensory symptoms suggestive of small-fiber dysfunction; conventional sensory NCS were preserved, as would be expected if large fibers were spared; and an independent sudomotor measure was abnormal. Taken together, these findings provided a coherent alternative physiological framework that better accounted for the sensory component of his presentation [3,5].

If definitive etiological classification were required, skin punch biopsy with IENFD quantification would have strengthened the diagnosis. Skin biopsy provides direct structural assessment of cutaneous sensory fibers and can additionally provide information regarding autonomic innervation [8,11]. The absence of such confirmation is therefore appropriately acknowledged as a limitation rather than compensated for by overinterpreting the Sudoscan result.

Peripheral nerve hyperexcitability as an overlapping mechanism

The patient's description of muscular tension, twitching, warmth, and symptomatic relief with exercise also raises the possibility of an overlapping peripheral nerve hyperexcitability phenotype. CFS occupies the milder end of the peripheral nerve hyperexcitability spectrum and can manifest with fasciculations, cramps, muscular aching and stiffness in the absence of progressive motor impairment [12]. In the seminal description by Tahmoush et al., patients demonstrated muscle aching, cramps, stiffness, fasciculations, and electrophysiological evidence of peripheral nerve hyperexcitability; mild CK elevation was also present in some patients [12].

Our patient did not demonstrate the characteristic antibody profile of a recognized autoimmune peripheral nerve hyperexcitability syndrome; anti-VGKC antibodies and anti-GAD65 antibodies were negative. However, antibody negativity does not convert the phenotype into ALS, nor does it exclude a nonimmune or seronegative hyperexcitability state. Therefore, peripheral nerve hyperexcitability is considered a plausible overlapping mechanism, rather than assigning CFS or Isaacs syndrome as a definitive diagnosis.

This distinction may also help reconcile apparently disparate aspects of the presentation. Small-fiber dysfunction could account for the cutaneous hypersensitivity, warmth and sudomotor abnormality, while chronic radiculopathy and focal neurogenic abnormalities could account for selected EMG changes. Superimposed peripheral motor axonal hyperexcitability could then contribute to fasciculations, muscle tension and twitching without requiring a single diffuse degenerative process to explain every manifestation.

Mild hyperCKemia

The fluctuating CK elevation also initially complicated the diagnostic evaluation. CK values ranged from 382 to 659 U/L, raising consideration of a primary myopathic process. However, CK lacks disease specificity and must be interpreted in relation to clinical weakness, electrophysiological morphology, other muscle enzymes, and disease trajectory.

In this patient, the modest CK elevation occurred without progressive proximal weakness, a myopathic EMG pattern, or elevation of aldolase. A comprehensive myositis antibody panel was negative, and there was no convincing clinical syndrome of inflammatory myopathy. The fluctuation from 659 U/L to 382 U/L and subsequently 549 U/L also did not suggest a steadily progressive destructive myopathy. Mild CK elevation has previously been described in peripheral nerve hyperexcitability syndromes; two of the nine patients in the original CFS series had mildly elevated CK [12]. Repetitive muscle activity, cramping, exercise, chronic neurogenic change, and other nonmyopathic mechanisms may therefore plausibly contribute to modest hyperCKemia.

The CK finding consequently provides another example of the central lesson of this case: an isolated abnormal investigation should not supersede the clinical phenotype. In the absence of weakness, normal aldolase, negative myositis serology, and a predominantly neurogenic rather than myopathic electrophysiological pattern, inflammatory myopathy became increasingly unlikely.

Extensive negative testing

The extensive investigation for alternative causes was valuable primarily because it progressively narrowed the differential. Negative anti-acetylcholine receptor antibodies and an incompatible phenotype argued against myasthenia gravis; negative anti-GAD65 and anti-VGKC antibodies reduced support for recognized antibody-associated hyperexcitability syndromes; the paraneoplastic neuronal panel was unrevealing; heavy-metal screening excluded several toxic mimics; HTLV testing was negative; lactate and pyruvate did not suggest a mitochondrial metabolic process; and comprehensive myositis serology, ANCA, extractable nuclear antigen (ENA), anti-dsDNA, and complement assessment provided no compelling evidence for systemic inflammatory or connective-tissue disease.

Nevertheless, a collection of negative investigations cannot itself establish SFN. The diagnosis in this patient emerged principally from positive clinical and physiological concordance: sensory hypersensitivity, abnormal warmth perception, preserved large-fiber conduction, sudomotor dysfunction, absence of progressive motor impairment, localization rather than dissemination on repeat EMG, and subsequent improvement with neuropathic therapy.

The isolated β2-glycoprotein I IgM positivity was also interpreted cautiously. In the absence of persistent positivity and appropriate clinical criteria, this finding was insufficient to establish antiphospholipid syndrome. Its coexistence with cutaneous vascular-appearing changes warranted follow-up, but it was not considered an adequate unifying explanation for the neurological presentation.

Why the therapeutic response matters

The patient's approximately 80% improvement after six weeks of gabapentin-based therapy provides an important clinical outcome, although it must not be used circularly to prove the diagnosis. Gabapentin reduces neuronal excitability principally through interaction with the α2δ subunit of voltage-gated calcium channels and is widely used for symptomatic treatment of neuropathic sensory disorders. Improvement is therefore biologically compatible with a neuropathic or hyperexcitability mechanism, but it is not specific to SFN.

The therapeutic response is nevertheless meaningful in the context of the complete trajectory. A patient initially facing the possibility of an inexorably progressive neurodegenerative disease remained objectively strong and functionally intact, had repeat electrophysiology that did not support neurodegeneration, and subsequently experienced major symptomatic improvement with neuropathic treatment. Such improvement is considerably more coherent with a symptomatic neuropathic/hyperexcitability syndrome than with the expected trajectory of untreated progressive motor neuron degeneration.

Vitamin E was administered concurrently; however, in the absence of documented vitamin E deficiency, its independent contribution to the clinical improvement cannot be determined. The improvement should therefore principally be reported as an outcome following gabapentin-based symptomatic therapy, rather than attributed mechanistically to vitamin E.

The danger of diagnostic anchoring

Perhaps the most consequential aspect of this case is diagnostic anchoring. Once an electrophysiological report contains the phrase “diffuse motor neuron disease,” subsequent symptoms may easily be interpreted through that diagnostic lens. Fasciculations become evidence of ALS; mild CK elevation becomes evidence of muscle destruction; and subtle asymmetry becomes evidence of progression. Yet the same findings can have entirely different significance when viewed against an eight-year history of preserved motor function.

The psychological implications are considerable. Patients referred for fasciculations frequently fear ALS, and Montalvo et al.'s BFS cohort notably included patients referred because of suspected ALS or because they themselves sought evaluation owing to concern about the disease [2]. Their observation that minor neurogenic EMG abnormalities can remain stable over years is particularly important because it demonstrates that even the combination of fasciculations and some chronic neurogenic change is not synonymous with progressive motor neuron degeneration [2].

The appropriate response to discordance is therefore neither to disregard an abnormal EMG nor to allow it to override the neurological examination. Rather, it is to interrogate the discordance itself. In our patient, asking when the symptoms truly began proved as diagnostically valuable as many of the subsequent laboratory investigations. An eight-year history without progressive weakness fundamentally changed the pre-test probability of ALS. Recognition that tremor was more prominent clinically than fasciculation, identification of positive sensory phenomena, documentation of preserved gait and power, and demonstration of sudomotor dysfunction further shifted the diagnostic framework. Repeat EMG then provided objective confirmation that a generalized neurodegenerative process had not emerged.

Clinical implications

This case therefore sits at the intersection of three common diagnostic problems: overinterpretation of fasciculations, overreliance on electrophysiology without longitudinal clinical correlation, and under-recognition of small-fiber sensory/autonomic dysfunction when conventional nerve conduction studies are normal. The literature supports each of these cautions. BFS can remain benign despite minor neurogenic EMG abnormalities [2]; SFN can produce substantial sensory and autonomic symptoms while routine NCS remains normal [3,9]; and objective small-fiber assessment requires methods distinct from standard large-fiber electrophysiology, with skin biopsy and IENFD quantification providing structural evidence of small-fiber pathology [8,13-15].

The case should not be interpreted as demonstrating that small-fiber dysfunction itself generated every abnormality on the original EMG. A more biologically plausible interpretation is that multiple nondegenerative processes coexisted: small-fiber/sudomotor dysfunction accounted for the prominent sensory phenotype; chronic lumbosacral radiculopathy and focal ulnar neurogenic abnormalities accounted for much of the conventional electrophysiological pathology; and an additional hyperexcitability component may have contributed to fasciculations, muscle tension and twitching. The diagnostic error would have been attempting to force these heterogeneous findings into a single progressive motor neuron disease simply because that diagnosis appeared capable of explaining the fasciculations and neurogenic motor-unit changes.

The patient's subsequent clinical course supports this integrated interpretation. The repeat EMG did not demonstrate progressive generalized denervation, motor function remained preserved, and symptoms improved markedly with neuropathic therapy. Collectively, these findings make an evolving generalized motor neuron disorder substantially less compelling and illustrate the value of longitudinal clinicophysiological reassessment.

Several limitations should be acknowledged. Skin biopsy with intraepidermal nerve-fiber density quantification was not performed; consequently, the findings support small-fiber dysfunction but do not establish biopsy-confirmed SFN. Muscle MRI and broad genetic testing for hereditary myopathies or myoneuropathies were also not undertaken. Although such investigations may be considered if objective weakness, progressive hyperCKemia, a myopathic electrophysiological pattern, or other suggestive features emerge, the prolonged preservation of motor function, normal aldolase, negative myositis evaluation, and predominantly neurogenic rather than myopathic repeat electrophysiology did not provide a compelling indication for these investigations during the present evaluation. Rare hereditary neuromuscular disorders therefore cannot be exhaustively excluded.

Learning points

Electromyographic abnormalities should always be interpreted within the clinical phenotype and disease trajectory, as suspected ALS requires progressive motor impairment. Prolonged preservation of strength and function should prompt diagnostic reassessment. Fasciculations and neurogenic EMG changes are not synonymous with ALS and may occur with radiculopathy, focal neuropathy, and peripheral nerve hyperexcitability. Prominent sensory symptoms despite normal routine nerve conduction studies should raise consideration of small-fiber dysfunction and the need for dedicated small-fiber assessment. When the initial electrophysiological findings and longitudinal clinical course remain discordant, repeat EMG can be diagnostically decisive by distinguishing progressive neurogenic dissemination from stable or anatomically localized abnormalities.

## Conclusions

This case illustrates the importance of longitudinal clinicophysiological correlation when an abnormal EMG raises concern for motor neuron disease, but the clinical phenotype and disease trajectory remain discordant. Although the initial fasciculations and multifocal neurogenic abnormalities appropriately prompted evaluation for ALS, the prolonged absence of progressive motor impairment, preserved strength and function, and localization rather than generalized progression on repeat electrophysiological assessment argued strongly against an evolving generalized motor neuron disorder. Prominent sensory manifestations, preserved large-fiber sensory conduction, and abnormal sudomotor testing supported coexisting small-fiber/autonomic dysfunction, although confirmatory structural assessment with intraepidermal nerve-fiber density was not performed. The overall presentation was therefore more consistent with overlapping nondegenerative neurological processes, including radiculopathic, small-fiber/autonomic, and possible hyperexcitability components, than with a single progressive motor neuron syndrome. This case emphasizes that an abnormal EMG should initiate clinicophysiological reconciliation rather than supersede the longitudinal clinical phenotype.
